# Sex and interferon gamma signaling regulate microglia migration in the adult mouse cortex in vivo

**DOI:** 10.1073/pnas.2302892120

**Published:** 2023-07-10

**Authors:** Roobina Boghozian, Sorabh Sharma, Kamal Narayana, Manjinder Cheema, Craig E. Brown

**Affiliations:** ^a^Division of Medical Sciences, University of Victoria, Victoria, BC V8P 5C2, Canada; ^b^Department of Psychiatry, University of British Columbia, Vancouver, BC V6T 2A1, Canada

**Keywords:** microglia, migration, interferon, sex differences, stroke

## Abstract

Although microglia possess the unique ability to migrate, whether mobility is evident in all microglia, is sex dependent, and what molecular mechanisms drive this, is not well understood in the adult brain. Using longitudinal in vivo two-photon imaging of sparsely labeled microglia, we find a relatively small population of microglia (~5%) are mobile under normal conditions. Following injury (microbleed), the fraction of mobile microglia increased in a sex-dependent manner, with male microglia migrating significantly greater distances toward the microbleed relative to their female counterparts. To understand the signaling pathways involved, we interrogated the role of interferon gamma (IFNγ). Our data show that in male mice, stimulating microglia with IFNγ promotes migration whereas inhibiting IFNγ receptor 1 signaling inhibits them. By contrast, female microglia were generally unaffected by these manipulations. These findings highlight the diversity of microglia migratory responses to injury, its dependence on sex and the signaling mechanisms that modulate this behavior.

## Significance

Microglia possess the unique ability to migrate in the mature brain, but the factors that govern this are poorly understood. Here, we show that while the population of mobile (“patrolling”) microglia is small under normal conditions, it can be rapidly expanded following injury. Microglia mobility after injury was dependent on biological sex, such that microglia from male mice were more likely to migrate, and they migrated greater distances toward the site of injury compared to their female counterparts. This migratory ability was regulated by interferon gamma signaling in males, but not females. Our findings highlight the diversity of factors that regulate microglia migration and add to emerging data that male and female brains have different immunological responses to injury.

Microglia are highly dynamic, innate immune cells that reside within the brain (1–3). Microglia movements can be categorized as those involving their processes (“process motility”) or the whole cell (“cell mobility or migration”). Under normal conditions, microglia continually rearrange their processes to scan the local microenvironment (4, 5). Following injury, microglial processes grow toward and engulf cellular debris, referred to as process chemotaxis (6, 7). However, microglia also possess the capacity to move to new locations or migrate (8). In early development, microglia derived from the yolk sack migrate into the brain (9), usually along nascent brain vasculature to populate their respective territories (10, 11). As the brain matures, there is some ambiguity regarding the extent to which microglia mobilize under normal/homeostatic conditions. For example, some longitudinal imaging studies report that microglia are very long-lived (median life-time over 15 mo) and stable in their somatic position during adulthood (10, 12–14). Other studies using fluorescent fate mapping or imaging have shown that a fraction of cells appeared in new positions. These changes in position were largely attributed to a balanced combination of cell proliferation and death (12, 15–17). Some instances of cell body movements were noted although on a very limited scale (18, 19).

The mobility of microglia toward sites of injury or toxic proteins in neurodegenerative disease, could be important for mollifying toxicity and resolving injury (20–24) but could also contribute to the spread of toxic proteins (e.g., Amyloid beta) (25) and pruning of synapses during development (26–29). Indeed, microglia migration has been noted by their clustered appearance around sites of injury (15, 30, 31). For example, Cx3Cr1-eGFP labeled microglia/macrophages show directed movements toward a hemorrhage in the first 48 h (14) or millimeters away from an ischemic stroke over weeks (32). Although these studies have been very informative, they have relied on transgenic mice that express a fluorescent reporter in most, if not all microglia. Thus, unambiguously tracking individual cell movements was nearly impossible, especially if a cell were to traverse a long distance in a short period of time, or if cells were to cluster in one spot, which invariably occurs after focal injury. Furthermore, since the transgenic mice used in these studies also label brain resident macrophages or infiltrative leukocytes (33), attributing movements specifically to microglia was not conclusive. Thus, there has been a paucity of quantitative data tracking migratory behaviors in individual microglia after injury.

Although we have a better understanding of the molecular mechanisms that regulate microglial surveillance and process chemotaxis (34, 35), the pathways that govern migration, beyond the role of P2RY12 signaling (16), has been mostly limited to in vitro studies or in retina (36). Proinflammatory cytokines such as IFNγ, are a likely candidate given that IFNγ receptors are expressed on microglia and its signaling is induced by brain injury such as stroke or hemorrhage, where it regulates many facets of macrophage behavior (37, 38). In cell or slice cultures, IFNγ exposure triggers inflammatory cytokine expression, phagocytic activity, and increases mobility (39, 40). Interestingly, cultured microglial responses to IFNγ can differ by sex (41), where male cells exhibit greater motility in the trans-well assay than their female counterparts. Sex differences also exist in microglia responses to ischemic stroke and/or proinflammatory stimuli (42–46). Based on these studies, it stands to reason that IFNγ signaling and sex could regulate migratory behaviors of microglia in the mature brain.

Despite our increased understanding of microglial biology (47), important questions remain concerning microglia mobility in the mature mammalian brain. For example, what fraction of microglia are mobile after vascular injury and are all cells equally capable of moving? How far can these cells travel per day? Is mobility affected by sex? What molecular mechanisms regulate mobility in the mature brain? In the present study, we tracked the mobility of individual cells using an inducible, microglia-specific cre-driver mouse. Our experiments show that while the population of mobile microglia is quite small under normal conditions, it can be rapidly expanded following injury. Further, we reveal that mobility was strongly influenced by biological sex and IFNγ signaling. Collectively, these results provide information about the factors (injury, sex, cytokine signaling) that regulate microglia mobilization in the mature brain.

## Results

### Methodological Considerations for Tracking Mobile Microglia In Vivo.

For the sake of clarity, the terms microglial “mobility or movements” herein refers to the movements of microglia cell bodies to new positions, not the movement of processes. To quantify microglial mobility in vivo, we had to first define what distance would constitute a true movement. Cells within the living brain can be pushed or displaced small distances for a number of reasons (changes in large vessel diameter, natural contortion of the brain) and repeated 3-dimensional measurements possess an intrinsic degree of error. Therefore, we measured the distance between GFP-labeled VIP interneurons in the cortex and a fiducial landmark at 12-h intervals in mice lightly anesthetized with isoflurane (*SI Appendix*, Fig. S1). Since fully differentiated neurons in the adult mouse cortex do not migrate, this allowed us to estimate measurement error in vivo. By tracking 206 neurons in three mice, we found the average displacement of cells was 1.84 ± 1.45 µm for 0 to 12 h and 1.61 ± 1.53 µm for 12 to 24 h (*SI Appendix*, Fig. S1). To minimize the possibility of false positive detection of cell “movement”, we determined that any distance ≥7.46 µm per 12-h period (four SD above mean distance derived from both 12-h timepoints) represented true cell movement. This threshold formed the basis for what we defined as a “mobile” cell.

**Fig. 1.**
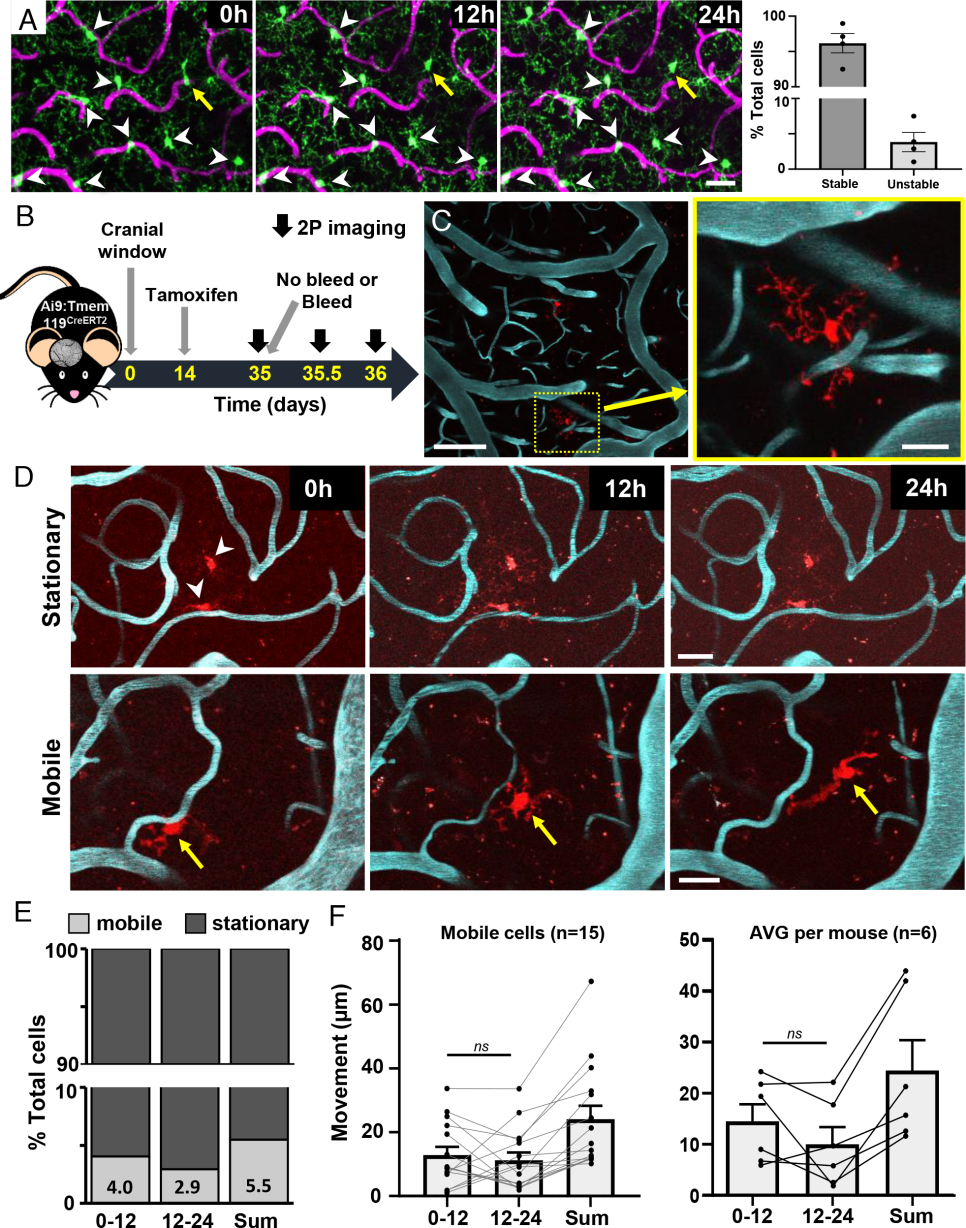
A small population of microglia are mobile in the normal adult brain. (*A*) Time lapse two-photon maximum intensity projection images of Cx3cr1-eGFP-labeled microglia/macrophages and vasculature over 12-h intervals (*Left*). The % cells that were “stable” or “unstable” (i.e., those that moved, appeared, or disappeared) is shown on the right. (Scale bars, 20 µm.) (*B*) Schematic showing timeline of experimental procedures and imaging. (*C*) In vivo maximum intensity projection images showing sparse tdTomato-labeled microglia and FITC-labeled blood vessels in the somatosensory cortex. (Scale bars, 100 and 20 µm.) (*D*) Time-lapse in vivo images of stationary (top row) or mobile (bottom row) microglia over a 24-h period. (Scale bars, 20 µm.) (*E*) Fraction of mobile or stationary microglia (out of 272 cells, n = 9 mice, 5 male and 4 female) imaged over 0 to 12 h, 12 to 24 h, or the sum of each window. Any microglia that moved >7.46 µm in a 12-h period was considered “mobile”. (*F*) Distance moved for each mobile microglia (*Left*) or the average distance per mouse (*Right*). Two-tailed paired *t* test indicated no difference between 0 to 12-h and 12 to 24-h periods in distance traveled per cell (t_(14)_ = 0.59, *P* = 0.57), or per mouse (t_(5)_ = 1.48, *P* = 0.20). Data are mean ± SEM.

To track the migration of microglia in adult mice, we first utilized heterozygous Cx3cr1-eGFP reporter mice (33), as described in previous studies that reported somatic movements (16, 18). These mice were implanted with a craniectomy-based cranial window and were allowed to recover at least 5 wk before imaging. We should note that thin skull imaging windows were also prepared although in our hands, this procedure often acutely disrupted superficial blood vessels and was associated with suboptimal signal to noise for deeper imaging. Consistent with previous imaging studies (16, 18), we found that the majority of putative microglia remained in the same position over a 24-h period (96.2% were “stable”, Fig. 1*A*). However, a small fraction of cells appeared to either move a short distance (see yellow arrow in Fig. 1*A*), appeared or disappeared (termed “unstable” microglia; move: 3.1% vs. new: 0.59% vs. lost: 0.24%; see yellow arrows in *SI Appendix*, Fig. S2*A*). Unfortunately, because the density of cell labeling was high, the shape of cells changed constantly and both microglia and macrophages express eGFP; we could not be sure if cells that changed (say appeared or disappeared) were microglia that moved a long distance or perhaps were infiltrative macrophages. Furthermore, we could not track single-cell movements after injury when cells rapidly cluster around a focal injury site (*SI Appendix*, Fig. S2*B*).

To track the movements of individual cells, we crossed the tamoxifen inducible and microglia-specific Tmem119-CreERT2 mouse line with cre-dependent tdTomato reporter mice (Ai9). Consistent with its original description (48), low-dose injection of tamoxifen 3 wk before the start of imaging yielded very sparse fluorescent labeling of microglia (2.84 ± 2.9% of total microglia population, based on 52 areas in 16 mice) in the adult mouse brain (Fig. 1 *B* and *C*). Furthermore, in the absence of tamoxifen, we did not find evidence of recombination (tdTomato labeled microglia), consistent with a recent study (49).

### A Small Population of Microglia Are Mobile under Normal Conditions.

Having optimized methodologies to track microglia in vivo, we then imaged their mobility over 1 d at time 0, 12, and 24 h (Fig. 1*B*). This imaging interval was determined based on pilot work indicating that considerable movement was possible in a 12-h period (especially after injury), thereby providing an interval of sufficient length to allow movement to occur, but not too long to cause us to lose track of individual cells. Under normal conditions, the majority of microglia were stationary (Fig. 1 *D* and *E*; 94.5% of 272 cells over 24 h), mirroring our estimate in Cx3cr1-eGFP mice. A small fraction of microglia clearly moved over this period, some upward of 40 to 60 µm (Fig. 1 *D* and *E*; 5.5%, 15/272 cells). On average, microglia classified as “mobile” moved equivalent distances for each 12-h period (~12.8 vs. 11.2 µm) for a mean of 24 µm over 24 h (Fig. 1*F*). We should note that since mice were imaged at the start of their light (8 am; 0 to 12 h) or dark cycle (8 pm, 12 to 24 h), we did not detect a circadian difference in movement per cell (Fig. 1 F, *Left*, two-tailed *t* test, t_(14__)_ = 0.59, *P* = 0.57) or per mouse (Fig. 1 *F*, *Right*, two-tailed *t* test, t_(5__)_ = 1.48, *P* = 0.20). Stratifying the movement of these mobile cells based on sex, did not reveal a significant effect of time or sex (two-way ANOVA, Time: F_(1, 26__)_ = 0.39, *P* = 0.54, Sex: F_(1, 26__)_ = 4.09, *P* = 0.053), although there was a trend toward reduced mobility in female cells (sum of movement over 24 h for males 28.94 ± 18.1 µm vs. 14.3 ± 4.03 µm in females). These results indicate that a small population of microglia are actively moving about in the uninjured brain.

### Sex Dictates Microglial Mobility in Response to Vascular Injury.

**Fig. 2.**
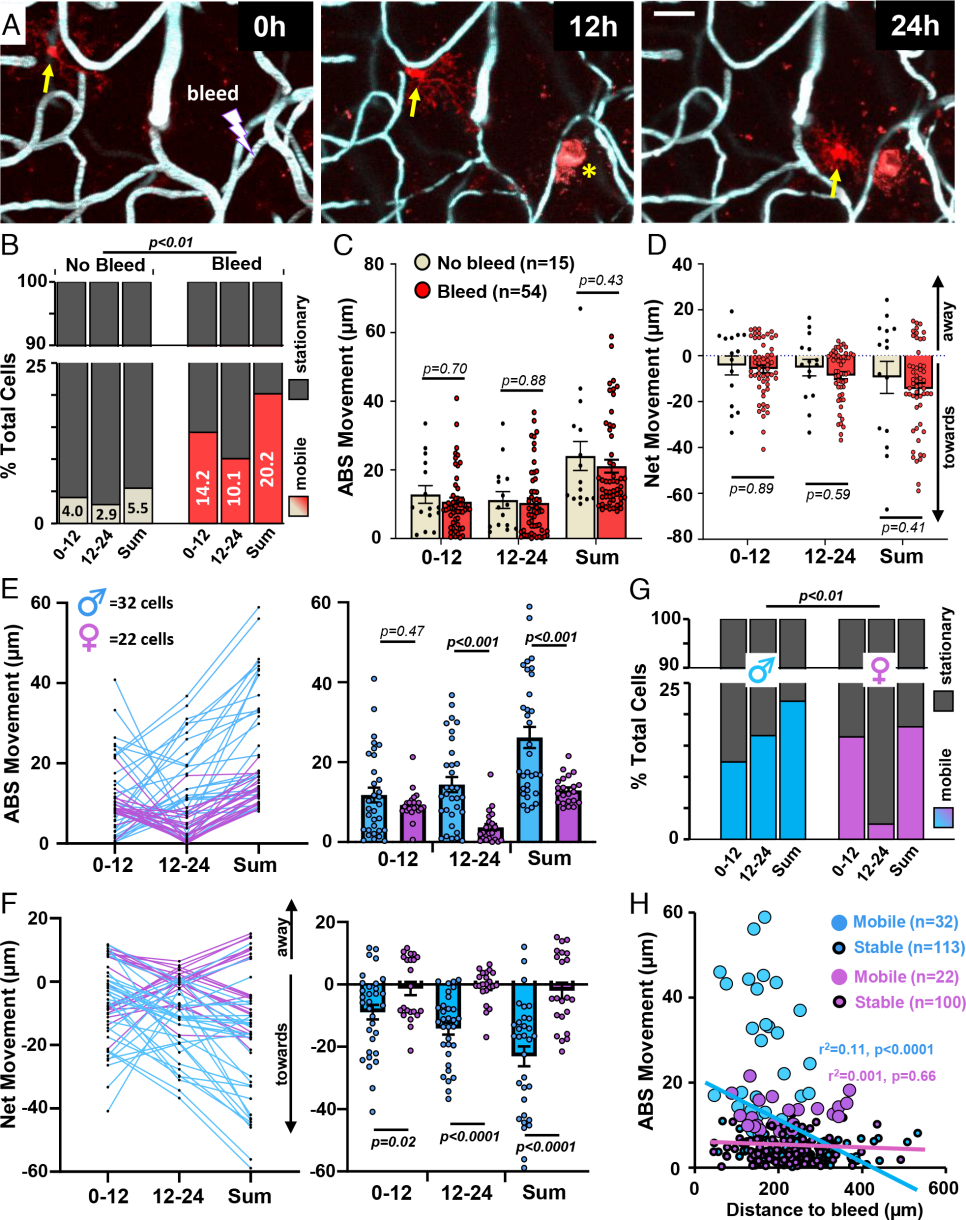
Sex-specific differences in microglial mobility after focal vascular injury. (*A*) In vivo two-photon images showing a microglia (yellow arrow) in a male mouse migrate toward a microbleed over a 24-h period. Note that vessel ablation leads to red-emitting autofluorescence at the injury site (yellow asterisks). (Scale bar, 20 µm.) (*B*) Fraction of mobile and stationary microglia imaged over a 24-h period under normal (no bleed; 272 cells from nine mice) or Bleed (267 cells from 13 mice) conditions, with data from both sexes pooled together. Chi-square analysis indicates the fraction of mobile microglia increased significantly after injury (χ2 = 109.9, *P* < 0.01). (*C*) Bar graphs show the absolute distance traveled by mobile microglia in No Bleed (15 cells) and Bleed (54 cells) conditions for each 12-h interval or the sum over 24 h, with data from both sexes pooled together. Data are mean ± SEM. (*D*) The net distance traveled by mobile microglia toward or away from the injury site (or an equivalent landmark in controls), with data from both sexes pooled together. Data are mean ± SEM. (*E*) Graphs show the absolute movement of each mobile microglia plotted over time (*E*, *Left*) or the group average (*E*, *Right*) after injury, stratified by sex (32 cells from seven males and 22 cells from six female mice). Data are mean ± SEM. (*F*) The net movement of each mobile microglia plotted over time (*F*, *Left*) or the group average (*F*, *Right*) after injury, stratified by sex. Data are mean ± SEM. (*G*) Fraction of mobile or stationary microglia for male and female mice after injury. Chi-square analysis indicates males had more mobile microglia after injury than female mice (χ2 = 9.33, *P* < 0.01). (*H*) Linear regression analysis in both male and female mice shows the relationship between how far a microglia moves over a 24-h period as a function of each cell’s initial distance from the bleed. Note the moderate and significant relationship in male but not female mice. Large open circles represent mobile microglia while smaller dots with dark outline represent stationary microglia. Statistical analysis for *C*–*F* was conducted with two-tailed unpaired *t* tests.

In order to stimulate microglia mobility, we assessed responses to a laser-induced cerebral microbleed (“CMB”; vessels 3.5 to 6 µm in diameter, see Fig. 1*B* for experimental outline). In vivo imaging showed that some microglia migrated long distances toward the bleed over a 24-h period (see example in Fig. 2*A*). As we did not find a significant effect of sex on mobility under normal conditions, we first analyzed mobility with data from both sexes pooled (see Fig. 2 *B–D*). When compared to uninjured controls, the induction of a CMB significantly increased the fraction of mobile microglia over 24 h (Fig. 2*B*, χ2 = 94.5, *P* < 0.01). However, to our surprise, the absolute distance of migration (Fig. 2*C*) and the net movement of mobile microglia toward the CMB (Fig. 2*D*) did not change significantly when compared to uninjured controls.

Since previous reports have shown that male microglia have a higher capacity to respond to ATP signals, display a more proinflammatory pattern of gene expression, and can appear morphologically different than females (44–46), we then stratified our data set by sex. This revealed that microglia from male mice migrated significantly greater absolute distances than female cells over the 24-h period, primarily in the later 12 to 24 h postbleed time window (Fig. 2*E*; two-way ANOVA, Sex: F_(1,104)_ = 15.6, *P* = 0.0001; Time: F_(1,104)_ = 0.81, *P* = 0.37; Sex x Time: F_(1,104)_ = 6.02, *P* = 0.02). Furthermore, microglia in male mice showed significantly greater net movement toward the CMB than female microglia (Fig. 2*F*; two-way ANOVA, Sex: F_(1,104)_ = 25.3, *P* < 0.0001; Time: F_(1,104)_ = 1.21, *P* = 0.27; Sex x Time: F_(1,104)_ = 1.99, *P* = 0.16). On average, male microglia migrated in a directional manner toward the microbleed (note negative values in Fig. 2*F*; *P* < 0.001 for one sample *t* tests conducted at each time point) whereas female cells showed little directional movement (*P* > 0.05 for one sample *t* tests conducted at each time point). The fraction of mobile microglia in male mice was significantly greater than in female mice (Fig. 2*G*; χ2 = 9.33, *P* < 0.01). And finally, as it was plausible that a cells’ relative distance from the CMB could influence mobility, we found in male mice that microglia closer to the bleed were more likely to migrate (Fig. 2*H*, r^2^ = 0.11, *P* < 0.0001), whereas in female mice, this relationship disappeared (Fig. 2*H*, r^2^ = 0.01, *P* = 0.37). We should note that the size of the bleed, based on the area of dye extravasation, was not significantly different between sexes (*SI Appendix*, Fig. S3; t_(23.9)_ = 0.93, *P* = 0.33). In addition to migratory behavior after injury (see additional examples in *SI Appendix*, Fig. S4), we also noted possible examples of microglia proliferation (*SI Appendix*, Fig. S5), although these were very infrequent events. Collectively, these results indicate that sex has a strong influence on mobility after vascular injury.

Given that sex and distance from injury were not the only variables at play in these experiments, we examined other factors that could influence mobility. First, we considered a cell’s depth from the cortical surface. Parsing microglia based on whether they were stationary or mobile did not reveal any systematic difference in cortical depth (*SI Appendix*, Fig. S6*A*), nor was there any significant relationship between movement distance and depth, even when stratifying cells by sex (*SI Appendix*, Fig. S6*B*). Given there is growing appreciation for the diversity of microglia, especially those that reside along capillaries (referred to as capillary associated microglia or “CAM”) that modulate blood flow (50, 51), we assessed mobility patterns in these cells. Our analysis of CAM vs. Non-CAM from both sexes did not show any significant differences in either the absolute or net movement of these cells after microbleed (*SI Appendix*, Fig. S6 C and D). Separating our analysis based on sex did not reveal any significant differences in movement between CAM vs. Non-CAM (*SI Appendix*, Fig. S6 C and D). In summary, our findings indicate that a cell’s depth below the cortical surface or whether it was associated with a capillary, does not significantly influence mobility.

### IFNγ Signaling Regulates Mobility after Injury in a Sex-Dependent Manner.

**Fig. 3.**
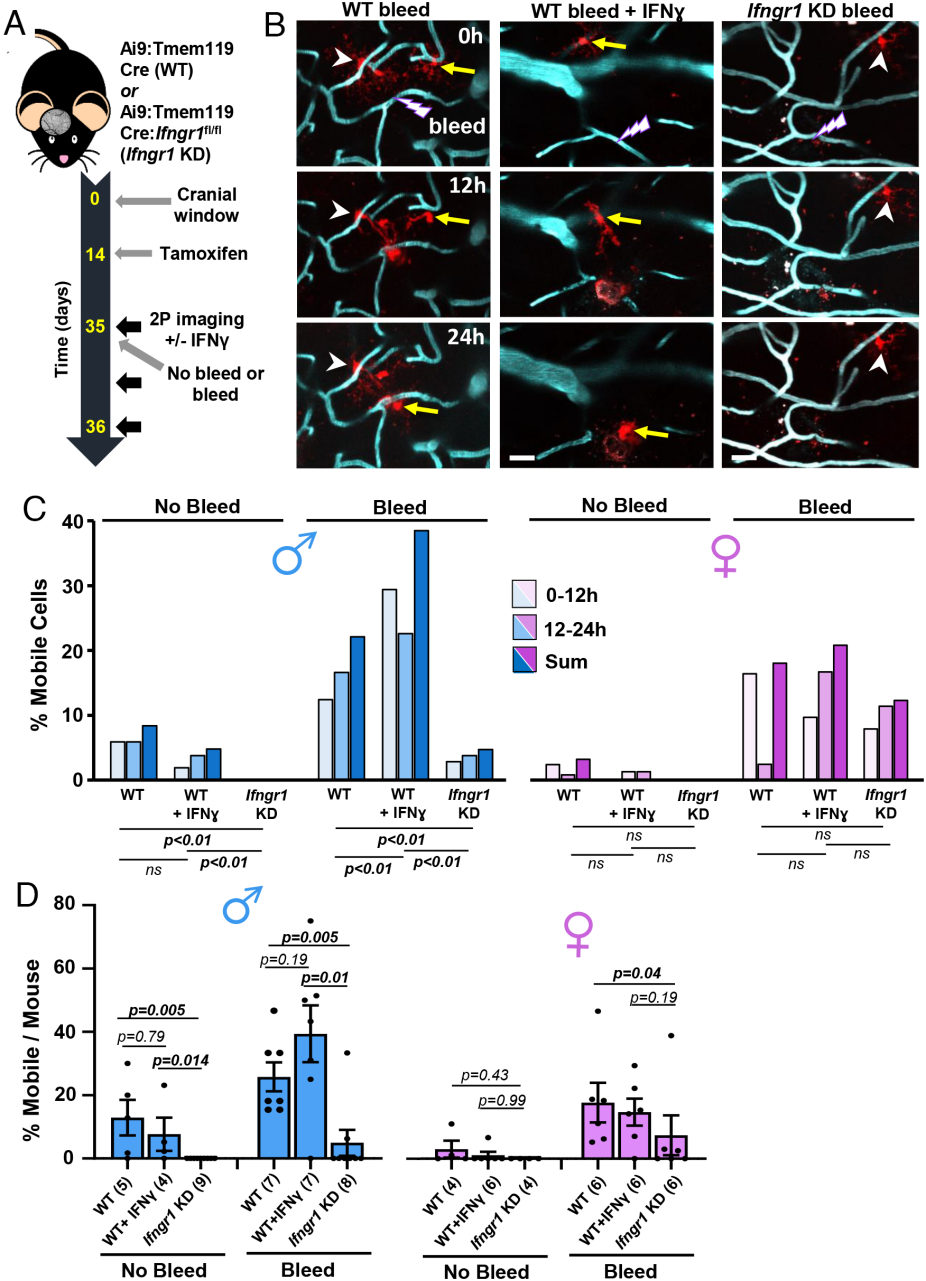
IFNγ signaling regulates the number of mobile microglia after microbleed in a sex-dependent manner. (*A*) Schematic showing timeline of experimental procedures and imaging. (*B*) In vivo two-photon images showing microglia movements in male mice after microbleed in wild-type (WT) controls, WT injected with IFNγ or microglia-specific knockdown of *Ifngr1* (*Ifngr1* KD). Mobile and stationary microglia are denoted by yellow arrow or white arrowhead, respectively. (Scale bar, 20 µm.) (*C*) Graphs show % of mobile microglia as a function of total cells sampled in each experimental group (MALES: WT no bleed and bleed: 119 and 145 total cells; WT+IFNγ no bleed and bleed: 105 and 252 total cells; *Ifngr1* KD no bleed and bleed: 122 and 106 total cells; FEMALES: WT no bleed and bleed: 153 and 122 total cells; WT+IFNγ no bleed and bleed: 75 and 72 total cells; IFNgR1 KD no bleed and bleed: 121 and 161 total cells). Chi-square analysis was used to compare groups. (*D*) Bar graph shows the percentage of mobile microglia for each mouse in each experimental group. The number of mice per group is indicated in parentheses. Three-way ANOVA was used to examine main effects of Sex, Bleed, and IFNγ status. *P* values were derived from two-tailed Mann–Whitney tests. Data are mean ± SEM.

Our next goal was to understand the molecular mechanisms that regulate microglial mobility. Previous studies have shown that IFNγ signaling is associated with a proinflammatory/disease related gene signature (39, 52), modulates microglial process chemotaxis in vivo (53); and preferentially stimulates migration in male microglia in vitro (41). To this end, we employed different approaches for enhancing or blunting IFNγ signaling in vivo. To stimulate IFNγ signaling, we intravenously injected recombinant mouse IFNγ or vehicle/control protein solution into Ai9:Tmem119cre (referred to as wild-type or “WT” controls) minutes before the induction of injury (Fig. 3*A*). We reasoned that IFNγ protein in the blood would easily leach into the brain parenchyma after vessel rupture and stimulate microglia. Furthermore, we could visually confirm the extravasation of fluorescent blood plasma after vessel injury (*SI Appendix*, Fig. S3). To knock down IFNγ signaling specifically within microglia, we crossed the Ai9:Tmem119cre line with IFNγ receptor 1 floxed mice (*Ifngr1*^fl/fl^), which previous studies have used to induce cell-specific knockdown of IFNγ signaling (54–56). This approach allowed us to simultaneously turn on a cre-dependent fluorescent reporter in a sparse population of microglia with cre-recombinase-dependent knockdown of *Ifngr1* (“*Ifngr1* KD”, see Fig. 3*B*).

Our statistical analysis of the fraction of mobile microglia per total cells (using chi-square analysis in Fig. 3*C*) or per mouse (using three-way ANOVA in Fig. 3*D*), indicated significant differences based on Sex, microbleed and IFNγ status (Sex: F_(1, 60__)_ = 6.64, *P* = 0.01, Bleed: F_(1, 60__)_ = 22.16, *P* < 0.0001, IFNγ: F_(2, 60__)_ = 7.613, *P* < 0.001), with strong trends for a Bleed x IFNγ (F_(2, 60__)_ = 2.56, *P* = 0.08) and Sex x IFNγ (F_(2, 60__)_ = 2.74, *P* = 0.07) interaction. Under normal conditions (i.e., no microbleed), we did not find a single mobile microglia in either male or female mice with cre-recombinase-dependent knockdown of *Ifngr1* (*Ifngr1 KD* male: nine mice, 122 cells imaged; *Ifngr1 KD* female: n = 4 mice, 121 cells; Fig. 3 *C* and *D*). Consequently, Male *Ifngr1* KD mice had significantly fewer mobile microglia than WT mice injected with IFNγ or control/vehicle solution (Fig. 3 *C* and *D*). However, since female mice tended to have fewer mobile microglia in general, there were no significant group differences between *Ifngr1* KD and WT mice (± injection IFNγ) in the % mobile microglia as a fraction of total cells sampled or % cells per mouse (Fig. 3 *C* and *D*). Not surprisingly, male and female WT mice intravenously injected with IFNγ (no microbleed condition) did not differ from WT controls given that one would expect relatively little stimulation of IFNγ signaling in microglia with an intact blood–brain barrier.

Following the induction of a microbleed, male microglial migratory responses (Fig. 3*B*) differed from females when IFNγ signaling was manipulated. Relative to WT controls, there was a significant increase in the total fraction of mobile microglia in male mice following microbleed when IFNγ signaling was stimulated, whereas knockdown of *Ifngr1* resulted in significantly fewer mobile microglia (Fig. 3*C*). Similar trends were observed for males when analyzing the % mobile cells per mouse (Fig. 3*D*). However in female mice, we did not find a significant increase in the total fraction of mobile microglia or % mobile cells per mouse when injected with IFNγ (Fig. 3 *C* and *D*). Examination of migratory responses in female *Ifngr1* KD mice after microbleed revealed little or no changes in the total fraction of mobile cells (Fig. 3*C*) or mobile cells per mouse (Fig. 3*D*). Thus, we conclude that IFNγ signaling appears to regulate microglia mobility in male mice but has relatively little effect in female mice.

**Fig. 4.**
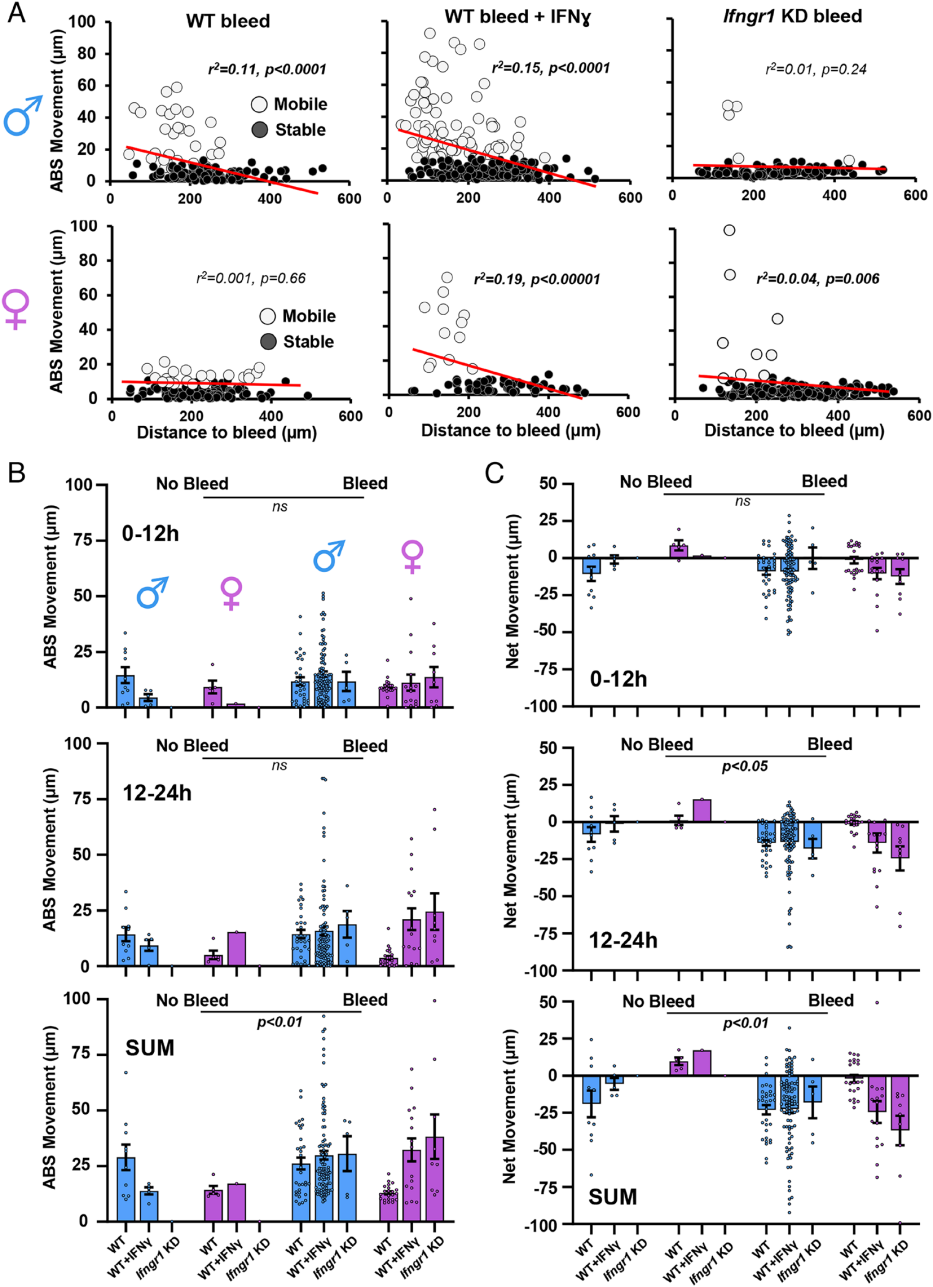
Effects of sex, IFNγ signaling, and injury on absolute and net microglial movements. (*A*) Linear regression analysis illustrates the relationship between the absolute movement of microglia over a 24-h period and the original distance from the injured vessel, for both male and female mice (top and bottom rows, respectively). Note that mobile cells are open circles while stable cells are filled circles. (*B*) Graphs show absolute movement of each mobile microglia in each experimental group over time. (*C*) Graphs indicate the net movement of mobile microglia. For data in *B* and *C*, a three-way ANOVA was used to examine main effects of Sex, Bleed, and IFNγ status at each time point. Data are mean ± SEM.

Next, we examined the influence of sex and IFNγ signaling on the relationship between mobility and location relative to the microbleed. In male WT mice injected with IFNγ protein or control solution, there was a moderate and highly significant relationship between microglial movement and their original distance from the microbleed, which was completely lost in male *Ifngr1* KD mice (top row in Fig. 4*A*). For female mice, the relationship between the initial distance from the bleed and absolute distance traveled, was absent or very weak in female WT and *Ifngr1* KD groups, respectively (bottom row in Fig. 4*A*; r^2^ values: 0.001 to 0.04), whereas a moderate relationship was evident in WT female mice injected with IFNγ (Fig. 4*A*; r^2^ = 0.19). Lastly, we plotted the absolute and net movement of all mobile microglia in each group (Fig. 4 *B* and *C*). A three-way ANOVA at each of the three time points did not indicate any significant main effects of Sex, IFNγ status or Sex x IFNγ status interactions on absolute or net movements for each time point examined (all *P* values > 0.05). However, there was a significant main effect of microbleed on the net movements of microglia at 12 to 24 h (F_(1,191)_ = 6.43, *P* = 0.01), as well as the sum of absolute (F_(1,191)_ = 7.19, *P* < 0.01) and net (F_(1,191)_ = 6.89, *P* < 0.01) movements (Fig. 4 *B* and *C*). These results suggest that although sex and IFNγ influence the fraction of microglia that become mobile after microbleed (especially for males), they do not affect how far these cells travel. By contrast, microbleeds when averaged across groups, do affect how far microglia move in a 24-h period, as well as their directionality once activated.

## Discussion

The extent to which microglia can mobilize in the mature mammalian cerebral cortex, has mostly been studied using histology or densely labeled transgenic mice. Furthermore, few studies have focused on the molecular mechanisms that regulate this behavior, at least in vivo. In the present study, we quantitatively assessed the mobility of individual microglia under normal and injury related conditions in mice with sparsely labeled microglia. Our experiments show that only a small fraction of microglia are mobile under normal conditions. After injury, the fraction of mobile microglia increased significantly, with some cells moving an impressive 60 to 100 µm in a 24 h period, especially when situated close to the injury. However, the capacity for some microglia to mobilize was strongly influenced by sex. For example, microglia in male mice were more likely to mobilize after injury, moved significantly greater distances, and showed greater directionality in movement when compared to microglia in female mice. In order to further understand the factors that regulate mobility, we probed the role of the proinflammatory cytokine IFNγ, in both male and female mice. Our experiments revealed that IFNγ signaling plays an important role in promoting microglia mobility, but primarily in male mice. Collectively, these findings reveal the heterogenous capacity for migration in microglia and the powerful influence that sex and IFNγ signaling play in this cellular behavior.

In agreement with previous studies, only a small fraction of microglia are mobile under normal conditions (16–18). However, the explanation for why so few mobilize remains speculative. Since microglia undergo cell death at low rates comparable to the percentage of cells that mobilize, it is conceivable that these cells move in order to repopulate territory vacated by dying or dead cells (16, 17). Dying cells would trigger the opening of NMDA receptors and release of chemoattractant signals like purines or lysophosphatidylcholine, which previous work has implicated in cell migration (16, 57, 58). Purine release could also come from the stochastic opening of the blood–brain barrier, such as microbleeds that do occur in healthy brains. However, these rare events would seem unlikely to account for the ~5% of cells that move per day. Very recent work has shown that microglia are highly attracted to bouts of neural activity (59). For example, increased neural activity can trigger NMDA receptor activity and purine release (60), which stimulates microglia process envelopment and suppression of active neurons (61). Whether similar mechanisms control whole-body movements of microglia remains an open question for future studies.

Following the induction of a microbleed, we show increased mobilization of microglia that cluster around injury sites (14, 62). Of note, some cells could travel up to ~100 µm within a 24 h period, which is further than past studies, likely due to previous limitations noted with single-cell tracking. This finding reinforces the idea that some microglia retain a remarkable capacity for migration well into adulthood. Indeed, this has been noted in one previous study showing putative microglia can migrate away from an injury site (2 to 4 wk after injury), on the order of millimeters (32). Another finding from our study was that the recruitment of microglia was strongly dependent on their initial distance from the site of injury, with the majority of mobile cells residing within 300 µm (14). This dependence on distance implies that a diffusible, injury-related factor was contributing. Based on previous work examining microglial process chemotaxis, there are several plausible mechanisms. Local changes in blood flow (63), the release of purines from damaged cells next to the injury site (64), or fibrinogen from blood plasma, are established signals for stimulating microglial process chemotaxis toward injury (27, 31, 64). Microbleeds can also locally change neural activity, although usually this is associated with a suppression of activity (65). Given that microglia are attracted to increased neural activity (59, 61), this would seem less likely to explain mobility toward the injury. Local changes in electrolytes or purines could also alter THIK-1 channel activity which regulates microglia transition states between surveillance and injury induced chemotaxis, as well as the release of proinflammatory cytokines (35).

Gene expression studies have firmly established that microglia express IFNγ receptors (37) and up-regulate IFNγ response genes after injury or neurodegeneration (52). Vascular insults such as ischemia and hemorrhages rapidly mobilize IFNγ secreting leukocytes to sites of injury (14, 66). Proinflammatory cytokines like IFNγ can pass through the blood–brain barrier, especially if weakened by injury (67) and stimulate a reactive, amoeboid like microglial morphology, secretion of proinflammatory cytokines (39, 40, 68) and migration, at least in cell culture (40) and retina (36). In the mature brain, our previous imaging study showed that IFNγ could affect microglia process chemotaxis toward vascular injury (53). However, despite the likelihood that IFNγ signaling could regulate microglia migration, direct in vivo tests of this in the adult cortex were lacking. Here, we show that stimulating IFNγ signaling by doping the blood with recombinant IFNγ, up-regulates the fraction of mobile cells after injury but does not significantly alter the absolute or net distance microglia travel. Conversely, genetic knockdown of *Ifngr1* specifically within male microglia, blunted the fraction of microglia that could be mobilized, but not the overall distances they travel. These results suggest that microglia mobility is like a switch. Once they are stimulated to become mobile cells, the distance with which they move is no longer tightly regulated by these stimulants. Even in the absence of injury, microglia in *Ifngr1* knockdown were much less mobile, as we did not find a single mobile cell in 13 mice (nine male, four female). This finding suggests that IFNγ signaling may exert some tonic effect on mobility in the normal brain, perhaps through low-level expression of IFNγ in resident immune cells, or conceivably constitutive IFNγ receptor activity. Consistent with this idea, the Kipnis group (54) showed that IFNγ secreting cells in the meninges can regulate neural and social activity in healthy mice, presumably through diffusion of IFNγ in cerebrospinal fluid to neurons. In the context of the present work, immune cells that secrete IFNγ such as T cells and monocytes, which are normally present in the meninges or recruited to sites of injury (69, 70), could directly communicate with microglia to regulate mobility. Indeed, a fascinating study (71) showed that the recruitment of T cells after ischemic stroke, activated microglia in the peri-infarct region (i.e., deramified morphology) and induced gene expression associated with interferon signaling and chemotaxis.

Whether mobilization of microglia is of help or hindrance to normal brain function or repair after injury, is not well understood. We do know that inhibiting microglial process motility or depleting them altogether with CSFR1 inhibitors, generally exacerbates damage after various forms of brain injury (22, 23, 72–75). Whether mobility of the cells themselves is critical, requires more thorough investigation. Systemically stimulating IFNγ signaling after middle cerebral artery occlusion, which could also stimulate migration, exacerbates ischemic damage (66). Similarly, upregulation of IFNγ signaling in microglia after viral infection promoted synapse loss and cognitive deficits in mice (55). However since IFNγ has pleiotropic effects, one could not conclude that these damaging effects were due to enhancing mobility. In the absence of injury, a loss of migratory abilities could have interesting functional consequences. For example, an inability to mobilize could limit phagocytic activity and thus affect the pruning of synapses, which is presumably beneficial in normal brain development, but deleterious in aging and neuropathology (26, 28). While beyond the scope of the present study, future studies could manipulate IFNγ signaling to promote or limit microglial mobilization toward lesions associated with vascular injury or multiple sclerosis (76), and determine if they hold any therapeutic benefit. This more subtle approach might be preferable to microglial depletion approaches, which have the unintended consequence of facilitating the recruitment of potentially deleterious infiltrative macrophages (76–78).

A common thread through most of our experimental results was the dependence of migration on biological sex. Indeed, we found that microglia in male mice were significantly more likely to mobilize toward injury than female mice, and these cells traveled greater distances. Furthermore, stimulating or inhibiting IFNγ signaling had much greater effects on migration in microglia from male mice than females. These results suggest that microglia in male mice may be more primed to migrate in response to injury or inflammatory signals (79–81). For example, microglia from male mice are larger, more branched, display stronger ATP-evoked currents and are enriched with genes and proteins associated with inflammation and chemotaxis (41, 44–46). While sex differences in migratory abilities in the cortex have not been extensively studied in vivo (41), in vitro data show that male microglia can migrate greater distances than female microglia, when stimulated with IFNγ. Similarly, male brains show significantly higher levels of IFNγ when stimulated with LPS than female brains (82). These findings could explain in our study why microglia in male mice were much more affected by manipulations of IFNγ than female mice. At the very least, it suggests that female microglia rely on additional factors to become mobile. Why these sex-specific differences in response to microbleed or IFNγ exist, is unclear. The sex differences could be intrinsic to microglia or the substrates around them, which permit migration. For example, sex hormone signaling in microglia can partially account for microglial differences in spinal hyperexcitability and pain sensitivity (80), but less so for gene expression differences (44). Sex chromosomes can also independently influence microglia function and gene/protein expression either directly or indirectly, and in fact strongly modulate sensitivity to stroke damage in aged mice (83). There are also reported sex differences in the turnover of extracellular matrix components (84), and higher cortical gene expression of laminin and collagen in the female cerebral cortex (85). Moreover, males exhibit increased levels of matrix metalloproteinases (MMP-1,2,3 and 9) in blood serum after intracerebral hemorrhage whereas changes in females were minimal (86). Resolving direct or indirect sex-specific changes in mobile microglia could be enlightened by single-cell analysis of gene expression, although this would be technically challenging since one would need to identify which microglia were mobile.

Considering that sex influences the susceptibility and outcomes of neurological conditions associated with alterations in IFNγ signaling (e.g., multiple sclerosis, vascular dementias, stroke, etc.), our results showing sex differences in migratory responses to injury could have functional implications. Human studies are rather mixed but show that males diagnosed with cerebral amyloid angiopathy (87), mild cognitive impairment, or Alzheimer disease (88), have significantly more cerebral microbleeds than females (89), whereas females with small vessel disease have increased microbleed burden (90). In rodents, the picture appears more consistent where female mice exhibit more abundant microbleeds than males in mouse models of Alzheimer disease (91) or cerebral amyloid angiopathy (92). Of note, the neurological outcome of female mice appeared worse given they had greater impairments in working memory and lower levels of circulating cytokines, including IFNγ (92). While highly speculative at this time, lower levels of circulating IFNγ in female mice could limit mobility of microglia toward vascular injury and repair, thus exacerbating the deleterious effects of vascular injury. Given the paucity of data examining the influence of sex, IFNγ, and microglia mobility in disease susceptibility and outcome, more research is warranted.

## Materials and Methods

### Animals.

Adult male and female mice between 2 and 6 mo of age were used in this study. For labeling of microglia and monocyte-derived macrophages, experiments involved heterozygous CX3CR1^+/GFP^ mice (JAX# 005582) on a C57BL/6J background (JAX# 000664). For cre-dependent inducible expression of the tdTomato reporter and/or knockdown of *Ifngr*1, we utilized Ai9 reporter mice (B6.Cg-*Gt(ROSA)26Sor^tm9(CAG-tdTomato)Hze^*/J, JAX# 007909) crossed with an inducible microglia-specific cre driver line (*Tmem119^em1(cre/ERT2)Gfng^*/J, JAX# 031820). These Ai9:Tmem119^CreERT2^ mice were then crossed with floxed *Ifngr*1 mice (C57BL/6N-*Ifngr1^tm1.1Rds^*/J, JAX# 025394) and bred to homozygosity. All mice were housed in groups on a 12-h light/dark cycle in ventilated racks in a humidity (RH 40 to 55%) and temperature-controlled room (21 to 23 °C). Mice were provided food and water ad libitum. All experiments comply with the guidelines set by the Canadian Council on Animal Care and approved by the local university Animal Care Committee. Reporting of this work complies with ARRIVE guidelines.

### Surgical Procedures.

Mice of at least 2 mo of age, were anesthetized using isoflurane (2% for induction and 1.3% for maintenance) in medical air (80% N_2_, 20% O_2_) at a flow rate of 0.7 L/min. A temperature feedback regulator and rectal probe thermometer maintained animals’ body temperature at 37 °C throughout the procedure. After subcutaneous injection of lidocaine under the scalp, an incision was made along the midline. A custom metal ring (~1 g in weight, outer diameter 11.3 mm, inner diameter 7.0 mm, height 1.5 mm) was positioned over the right somatosensory cortex and secured to the skull with metabond adhesive. A circular area (diameter of ~4 to 5 mm) of skull within the metal ring was thinned using a high-speed dental drill, and ice-cold HEPES-buffered artificial cerebrospinal fluid (ACSF) was periodically applied to the skull for cooling purposes. Fine forceps were used to remove the thinned piece of skull and the exposed brain was kept moist with gel foam soaked in cold ACSF. A 5- or 6-mm coverslip (no. 1 thickness) was placed over the exposed brain and secured to the skull using cyanoacrylate adhesive. After the procedure, mice were injected with 0.03 mL 2% dexamethasone (i.p.) to reduce acute inflammation resulting from the procedure. Mice were monitored while they recovered under a heat lamp and then were returned to their home cage.

### Experimental Treatments and In Vivo Two-Photon Imaging.

Two to four wk after cranial window implantation, cre-recombinase-dependent expression of tdTomato reporter and/or knockdown of *Ifngr*1 was induced with an intraperitoneal injection of 0.5 mg Tamoxifen (Sigma #T5648) dissolved in corn oil (Sigma #T5648). If mice failed to show sufficient reporter expression 2 wk later, they were reinjected with the same dose of tamoxifen.

For two-photon imaging, mice were lightly anesthetized with isoflurane (2% for induction and 1% for maintenance in medical air), and then received 0.1 mL of either 1.5 to 3% fluorescein isothiocyanate–dextran (FITC) or Texas Red dextran (70 kDa, Sigma-Aldrich #46945 and Thermofisher D1830, respectively), to permit visualization of the cerebral vasculature. The metal ring on the mouse’s head was fixed into a custom imaging stage. High-resolution two-photon image stacks of the vasculature and GFP or tdTomato expressing microglia/macrophages were acquired in vivo using an Olympus FV1000MPE laser scanning microscope fed by a mode-locked Ti:Sapphire laser source (Mai Tai XF DeepSee, Spectra-Physics) and equipped with a water-dipping 20× objective lens [Olympus; numerical aperture (NA) = 0.95]. The laser was tuned to 900 nm for imaging Texas Red dextran and eGFP, while 945 nm was used for imaging FITC and tdTomato-labeled microglia. Emitted light was split by a dichroic filter (552 nm) before it was directed through band-pass filters (495 to 540 nm and 558 to 630 nm). Images were collected at 1.75-μm z-step intervals from the cortical surface to a depth of 75 to 200 µm, with each image covering an area of 635.3 × 635.3 μm (1024 × 1024 pixel sampling). In order to maximize sampling of sparsely labeled microglia, we typically imaged 2 to 5 different regions within each mouse, spaced at least 500 µm apart from each other. Capillaries that were 3 to 6 µm in diameter were targeted for laser-induced rupture. As previously described, we raster scanned a 4x4-µm region of interest centered on the capillary for 3 to 8 s using the femtosecond laser (805 nm, ~390 mW at back aperture, 10-µs pixel dwell time). The rupture of a cortical capillary was confirmed in real-time by the extravasation of fluorescently labeled blood plasma. Brightfield images of the brain’s surface and vascular landmarks were used to relocate imaging areas over time.

For modulating IFNγ signaling in vivo, we used two approaches. First for stimulating IFNγ signaling (66), we randomly assigned Ai9:Tmem119 cre mice to receive an intravenous injection of control solution or 0.05 mg/kg of recombinant IFNγ from mouse (Sigma #I4777), minutes before initiating the rupture of cortical capillaries and then 12 h later. Control solution injections consisted of injecting vehicle with or without a protein control (albumin or isotype control antibody, clone 2A3, Bio X Cell). Our analysis indicated that there were no significant differences in the fraction of mobile microglia or ABS migration distance between vehicle and control protein-injected mice (% mobile cells: two-tailed *t* test, t_(5__)_ = 0.75, *P* = 0.48; ABS movement Sum: two-tailed *t* test, t_(30__)_ = 0.89, *P* = 0.38), therefore data from these groups were pooled together under “WT bleed” group.

### Data Analysis.

In order to estimate error associated with three-dimensional measurements in vivo, we repeatedly imaged GFP-labeled VIP neurons in mice (*Vip^tm1(cre)Zjh^*/J, JAX# 010908) at 12-h intervals (*SI Appendix*, Fig. S1). Using Neurolucida software, we quantified the Euclidean distance from the center of each cell body to a fiducial vascular landmark in the center of the image. Performing this analysis in three mice with 206 neurons, yielded a mean error of 1.84 ± 1.45 µm and 1.61 ± 1.53 µm for 0 to 12 and 12 to 24-h measurements. To minimize any false-positive errors, we set the minimum migration cutoff at 4-SD above the mean error values to yield a minimum cutoff migration distance of 7.46 µm in a 12-h period.

Having established our criteria for detecting true migration, we then classified microglia as “mobile” or “stationary” based on whether their 3D position from a fiducial vascular landmark (typically a branch point) changed by more than 7.46 µm at 0 to 12 or 12 to 24 h. The sum of mobile cells represented all those that exceeded this criteria at either or both time intervals. Since cells could move toward or away from a vascular landmark or bleed site, movement was calculated as either the absolute value (thus indifferent to direction) or the net value relative to vascular landmark or bleed location. For the analysis of movement in CAM, we first determined whether the closest edge of a microglia soma was within 1.86 µm (three pixels) from the lumen of a flowing capillary or not. Based on this dichotomy, we than analyzed the absolute and net movement of each microglia across and within sexes. In order to estimate the extent of plasma extravasation after microbleed, we calculated extravasation area from single images collected immediately after the rupture that were then mean filtered (radius = two pixels) and thresholded using Huang’s method in Image J.

### Statistics.

Absolute and net distances traveled by mobile microglia were analyzed with two-way or three-way ANOVA using either time, injury (bleed vs. no bleed), Sex and IFNγ status (WT control vs. WT+IFNγ vs. Ifngr1 KD) as factors. Post hoc tests were adjusted using Sidak’s multiple comparisons tests. Chi-squared analysis was used to determine if the fraction of mobile cells differed between experimental groups. The “observed” values for each experimental group were compared to the “expected” values, which were derived from no bleed or male groups in Fig. 2, or WT group in Figs. 3 and 4. Linear regression analyses were used to determine the relationship between a cell’s initial distance from a microbleed and the absolute distance it traveled over 24 h after bleed. Nonparametric tests (two-tailed Mann–Whitney test) were used to compare groups when examining the % mobile cell per mouse. Unless otherwise stated, data are presented as mean ± SEM. Cutoffs for significant differences were as follows: *P < 0.05, **P < 0.01, ***P < 0.001.

## Supplementary Material

Appendix 01 (PDF)

## Data Availability

All study data are included in the article and/or *SI Appendix*.
